# Supplementary material for: Resolving the full spectrum of human genome variation using Linked-Reads
Source: Genome Res. 2019 Apr;29(4):635–45. doi: 10.1101/gr.234443.118 (PMC6442396; doi:10.1101/gr.234443.118)
Supplement: Supplemental Material [file supp_gr.234443.118_Supplemental_longranger-2.2.zip › longranger-2.2.tar.bz2!/lr2.2-public/lib/stan/cmdstan/stan/doc/api/html/structstan_1_1gm_1_1term__grammar.html]

Stan: stan::gm::term\_grammar< Iterator > Struct Template Reference


|  |  |
| --- | --- |
|  | Stan  2.5.0  probability, sampling & optimization |


- Main Page
- Related Pages
- Namespaces
- Classes
- Files

- Class List
- Class Index
- Class Hierarchy
- Class Members

All Classes Namespaces Files Functions Variables Typedefs Enumerations Enumerator Friends Macros Pages

- stan
- gm
- term\_grammar

Public Member Functions |
Public Attributes |
List of all members

stan::gm::term\_grammar< Iterator > Struct Template Reference

`#include <expression07_grammar.hpp>`

Inheritance diagram for stan::gm::term\_grammar< Iterator >:

|  |  |
| --- | --- |
| Public Member Functions | |
|  | term\_grammar (variable\_map &var\_map, std::stringstream &error\_msgs, expression\_grammar< Iterator > &eg) |
|  | |

|  |  |
| --- | --- |
| Public Attributes | |
| variable\_map & | var\_map\_ |
|  | |
| std::stringstream & | error\_msgs\_ |
|  | |
| stan::gm::expression\_grammar  < Iterator > & | expression\_g |
|  | |
| boost::spirit::qi::rule  < Iterator, std::vector  < expression >var\_origin),   whitespace\_grammar< Iterator > > | args\_r |
|  | |
| boost::spirit::qi::rule  < Iterator, std::vector  < expression >var\_origin),   whitespace\_grammar< Iterator > > | dims\_r |
|  | |
| boost::spirit::qi::rule  < Iterator, double\_literal(),   whitespace\_grammar< Iterator > > | double\_literal\_r |
|  | |
| boost::spirit::qi::rule  < Iterator,   boost::spirit::qi::locals  < bool >, expression(var\_origin),   whitespace\_grammar< Iterator > > | factor\_r |
|  | |
| boost::spirit::qi::rule  < Iterator, fun(var\_origin),   whitespace\_grammar< Iterator > > | fun\_r |
|  | |
| boost::spirit::qi::rule  < Iterator, integrate\_ode(var\_origin),   whitespace\_grammar< Iterator > > | integrate\_ode\_r |
|  | |
| boost::spirit::qi::rule  < Iterator, std::string(),   whitespace\_grammar< Iterator > > | identifier\_r |
|  | |
| boost::spirit::qi::rule  < Iterator, expression(var\_origin),   whitespace\_grammar< Iterator > > | indexed\_factor\_r |
|  | |
| boost::spirit::qi::rule  < Iterator, int\_literal(),   whitespace\_grammar< Iterator > > | int\_literal\_r |
|  | |
| boost::spirit::qi::rule  < Iterator, expression(var\_origin),   whitespace\_grammar< Iterator > > | negated\_factor\_r |
|  | |
| boost::spirit::qi::rule  < Iterator, expression(var\_origin),   whitespace\_grammar< Iterator > > | exponentiated\_factor\_r |
|  | |
| boost::spirit::qi::rule  < Iterator, expression(var\_origin),   whitespace\_grammar< Iterator > > | term\_r |
|  | |
| boost::spirit::qi::rule  < Iterator, variable(),   whitespace\_grammar< Iterator > > | variable\_r |
|  | |

## Detailed Description

### template<typename Iterator> struct stan::gm::term\_grammar< Iterator >

Definition at line 20 of file expression07\_grammar.hpp.

## Constructor & Destructor Documentation

template<typename Iterator >

|  |  |  |  |
| --- | --- | --- | --- |
| stan::gm::term\_grammar< Iterator >::term\_grammar | ( | variable\_map & | *var\_map*, |
|  |  | std::stringstream & | *error\_msgs*, |
|  |  | expression\_grammar< Iterator > & | *eg* |
|  | ) |  |  |

Definition at line 625 of file term\_grammar\_def.hpp.

## Member Data Documentation

template<typename Iterator >

boost::spirit::qi::rule<Iterator, std::vector<expression>var\_origin), whitespace\_grammar<Iterator> > stan::gm::term\_grammar< Iterator >::args\_r

Definition at line 44 of file term\_grammar.hpp.

template<typename Iterator >

boost::spirit::qi::rule<Iterator, std::vector<expression>var\_origin), whitespace\_grammar<Iterator> > stan::gm::term\_grammar< Iterator >::dims\_r

Definition at line 50 of file term\_grammar.hpp.

template<typename Iterator >

boost::spirit::qi::rule<Iterator, double\_literal(), whitespace\_grammar<Iterator> > stan::gm::term\_grammar< Iterator >::double\_literal\_r

Definition at line 56 of file term\_grammar.hpp.

template<typename Iterator >

std::stringstream& stan::gm::term\_grammar< Iterator >::error\_msgs\_

Definition at line 36 of file term\_grammar.hpp.

template<typename Iterator >

boost::spirit::qi::rule<Iterator, expression(var\_origin), whitespace\_grammar<Iterator> > stan::gm::term\_grammar< Iterator >::exponentiated\_factor\_r

Definition at line 104 of file term\_grammar.hpp.

template<typename Iterator >

stan::gm::expression\_grammar<Iterator>& stan::gm::term\_grammar< Iterator >::expression\_g

Definition at line 38 of file term\_grammar.hpp.

template<typename Iterator >

boost::spirit::qi::rule<Iterator, boost::spirit::qi::locals<bool>, expression(var\_origin), whitespace\_grammar<Iterator> > stan::gm::term\_grammar< Iterator >::factor\_r

Definition at line 63 of file term\_grammar.hpp.

template<typename Iterator >

boost::spirit::qi::rule<Iterator, fun(var\_origin), whitespace\_grammar<Iterator> > stan::gm::term\_grammar< Iterator >::fun\_r

Definition at line 69 of file term\_grammar.hpp.

template<typename Iterator >

boost::spirit::qi::rule<Iterator, std::string(), whitespace\_grammar<Iterator> > stan::gm::term\_grammar< Iterator >::identifier\_r

Definition at line 80 of file term\_grammar.hpp.

template<typename Iterator >

boost::spirit::qi::rule<Iterator, expression(var\_origin), whitespace\_grammar<Iterator> > stan::gm::term\_grammar< Iterator >::indexed\_factor\_r

Definition at line 86 of file term\_grammar.hpp.

template<typename Iterator >

boost::spirit::qi::rule<Iterator, int\_literal(), whitespace\_grammar<Iterator> > stan::gm::term\_grammar< Iterator >::int\_literal\_r

Definition at line 92 of file term\_grammar.hpp.

template<typename Iterator >

boost::spirit::qi::rule<Iterator, integrate\_ode(var\_origin), whitespace\_grammar<Iterator> > stan::gm::term\_grammar< Iterator >::integrate\_ode\_r

Definition at line 74 of file term\_grammar.hpp.

template<typename Iterator >

boost::spirit::qi::rule<Iterator, expression(var\_origin), whitespace\_grammar<Iterator> > stan::gm::term\_grammar< Iterator >::negated\_factor\_r

Definition at line 98 of file term\_grammar.hpp.

template<typename Iterator >

boost::spirit::qi::rule<Iterator, expression(var\_origin), whitespace\_grammar<Iterator> > stan::gm::term\_grammar< Iterator >::term\_r

Definition at line 110 of file term\_grammar.hpp.

template<typename Iterator >

variable\_map& stan::gm::term\_grammar< Iterator >::var\_map\_

Definition at line 34 of file term\_grammar.hpp.

template<typename Iterator >

boost::spirit::qi::rule<Iterator, variable(), whitespace\_grammar<Iterator> > stan::gm::term\_grammar< Iterator >::variable\_r

Definition at line 116 of file term\_grammar.hpp.

---

The documentation for this struct was generated from the following files:

- src/stan/gm/grammars/expression07\_grammar.hpp
- src/stan/gm/grammars/term\_grammar.hpp
- src/stan/gm/grammars/term\_grammar\_def.hpp


---

[ Stan Home Page ]

*© 2011–2014,
Stan Development Team.*
